# Successful analgesic treatment with continuous sacral epidural ethanol injection therapy for anal pain caused by multiple metastases of malignant pheochromocytoma

**DOI:** 10.1186/s40981-024-00760-x

**Published:** 2024-12-18

**Authors:** Takehito Sato, Shigeru Inoue, Ichiko Asano, Takahiro Ando, Yasuyuki Shibata

**Affiliations:** 1 Department of Anesthesiology, Nagoya University Hospital, Nagoya City, Aichi, 466-8550 Japan; 2 Division of Operation Room, Nagoya University Hospital, Nagoya, Aichi Japan

**Keywords:** Analgesic treatment, Anal pain, Ethanol injection

## Abstract

**Background:**

Anal and perineum pain caused by malignant tumor invasion is often difficult to control with opioids. Continuous sacral epidural ethanol injection therapy is less likely to cause bladder and rectal disturbances, making it a suitable treatment option for patients with preserved voiding function.

**Case presentation:**

A 45-year-old woman with multiple metastases of malignant pheochromocytoma suffered severe anal pain that worsened, especially when sitting, and was unresponsive to opioid rescue therapy.

With her NRS score of 9, a sacral epidural catheter was placed, and a continuous infusion of 2% lidocaine was administered overnight. This is followed by a 1.5mL bolus of ethanol and continuous ethanol administration at 2 mL/h. After administration, her anal pain decreased to approximately NRS 0–1, and she was subsequently discharged.

**Conclusion:**

We report successful pain control using continuous sacral epidural ethanol injection therapy in a patient with anal pain due to malignant pheochromocytoma metastasis.

## Background

Anal and perineal pain from malignant tumor invasion is often difficult to control with NSAIDs or opioids, significantly impacting patients’ quality of life [1, 2].

Continuous sacral epidural ethanol infusion involves placing a catheter in the sacral epidural space to continuously administer anhydrous ethanol, targeting only the perineal region [3]. Compared with other neuroleptic blocking techniques, it is less likely to cause bladder and rectal disturbances, making it a suitable option for patients with preserved voiding function [3].

We report a case in which a patient with multiple metastases of a malignant pheochromocytoma complained of anal pain that worsened while standing; good pain control was achieved with sacral epidural ethanol injection therapy.

## Case presentation

Written consent was obtained from the patient and her family for this case report. We also applied to our affiliated institution for approval of the off-label use of continuous sacral epidural block with absolute ethanol (Approval number: TF10013).

The patient was a 45-year-old woman (height: 170 cm; weight: 50 kg) who was receiving oral doxazosin mesylate (4 mg/day) and spironolactone (50 mg/day) to manage pheochromocytoma. Despite chemotherapy, her malignant pheochromocytoma had metastasized, and she had an estimated life expectancy of approximately 3 months.

She was admitted to the hospital for escalating pain, initially managed with 18 mg/day hydromorphone hydrochloride, which provided general pain relief but failed to alleviate persistent anorectal pain, especially aggravated while standing.

**Fig. 1:**
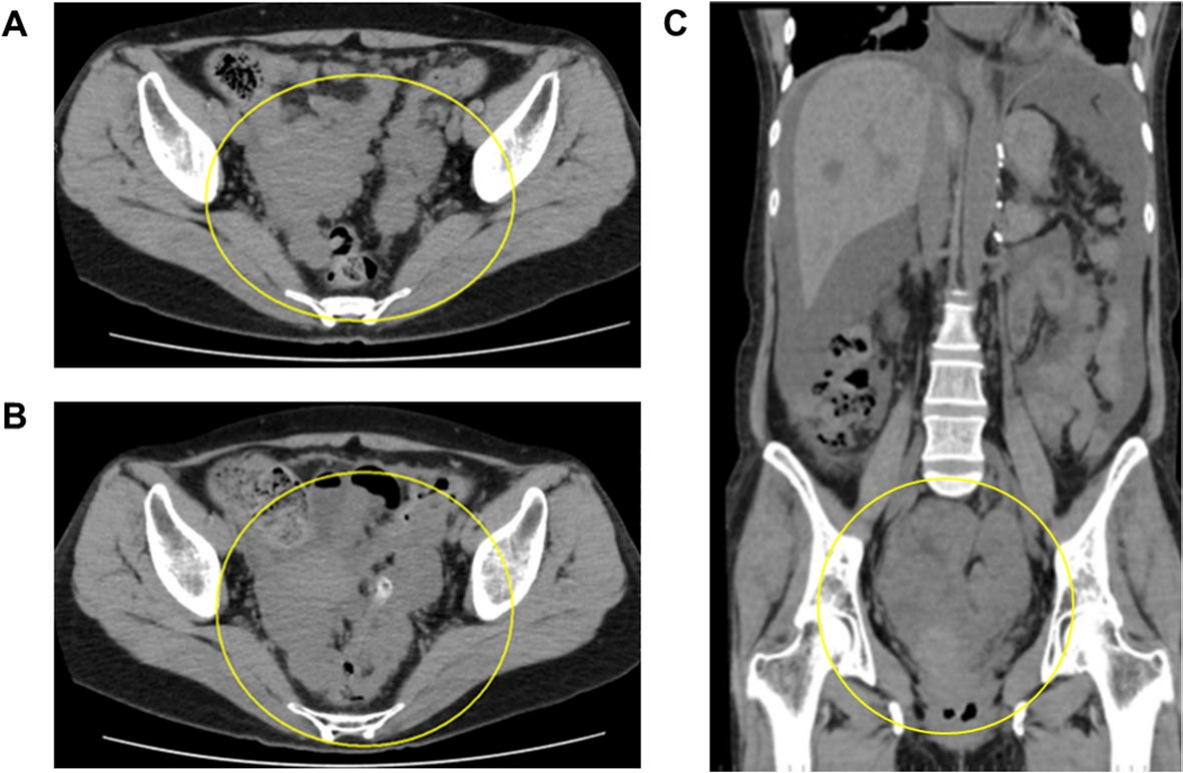
CT images. **A** One month before admission. **B** At the time of admission, CT showed that the progression of disseminated lesions in the pelvis was observed. **C** Coronal view at the time of admission: massive ascites and disseminated metastasis of pheochromocytoma surrounding the uterus

She complained of persistent anal and perineum pain, with a numerical rating scale (NRS) score of 8, worsened by standing and movement. The CT showed a large tumor mass near her uterus and anus, with suspected movement towards the anus in a standing position, causing exacerbation of her pain (Fig. 1).

**Fig. 2:**
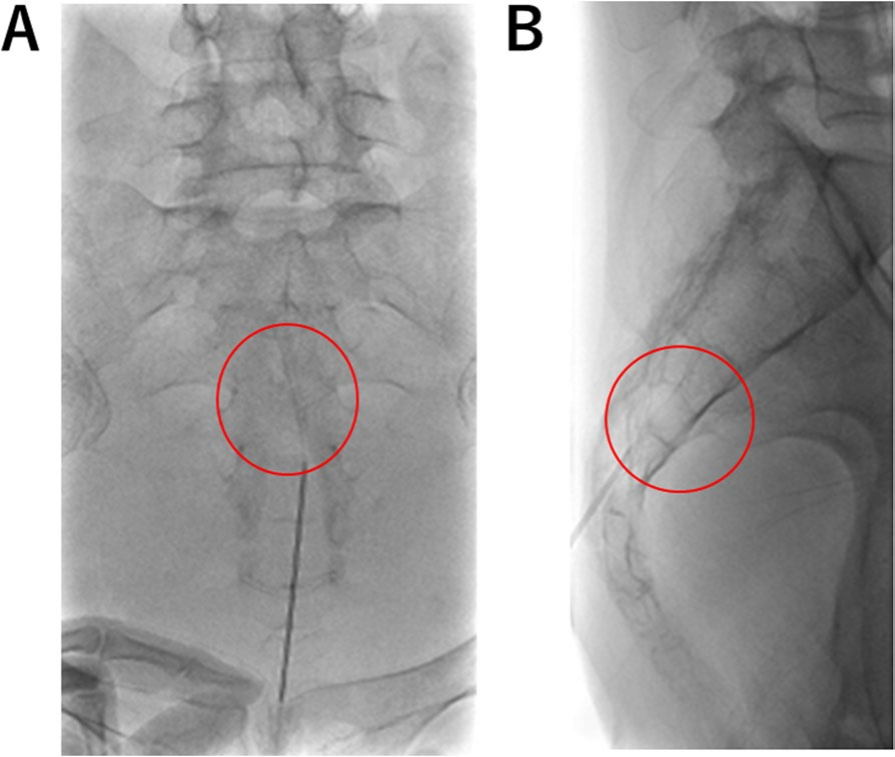
Fluoroscopic images. **A** Frontal view. **B** Lateral view. The tip of the epidural needle is at the S3/4 level

Given her symptoms, she was deemed an appropriate candidate for sacral epidural ethanol infusion therapy to relieve anal pain. An epidural catheter was inserted through the sacral hiatus under radiographic, fluoroscopic, and ultrasound guidance. After local anesthesia was administered subcutaneously with 1% lidocaine (Sandoz Pharma K.K., Tokyo, Japan) at the puncture site, an 18-gauge, 8.0-cm Tuohy needle (Hakko Co., Ltd., Nagano, Japan) was advanced into the sacral epidural space (Fig. 2).

**Fig. 3:**
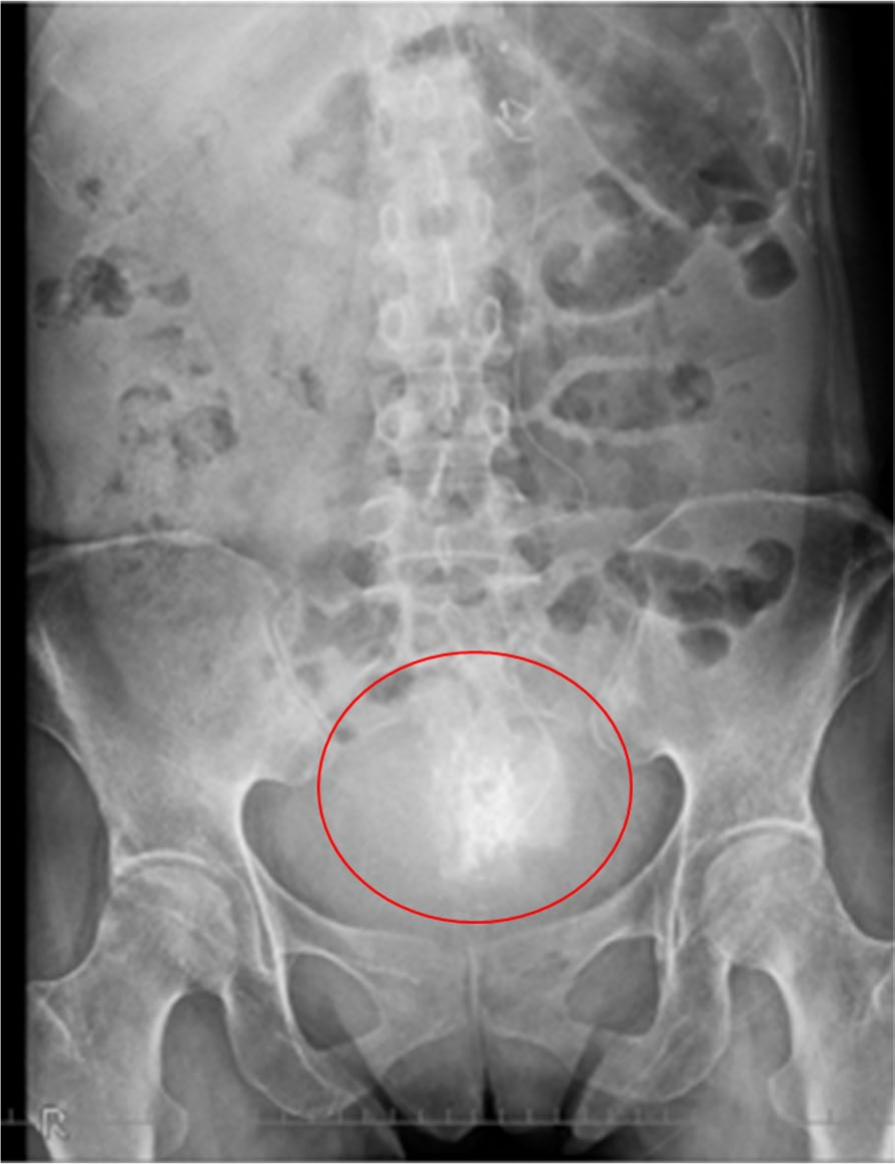
X-ray image injected with contrast agents the next morning after the sacral epidural catheter was placed. Contrast findings are seen around the S4/5 level

After placing the epidural catheter in the epidural space at the S2/3 level, confirmed by administering 1.5 mL of iohexol (Fig. 3), we performed a test block with 2 mL of 2% lidocaine and confirmed pain relief. Overnight, a continuous infusion of 2% lidocaine at 2 mL/h was administered. The next day, we slowly administered 1.5 mL of anhydrous ethanol (Viatris Inc., Tokyo, Japan) and then 2 mL/h of ethanol continuously. Treatment consisted of three cycles of continuous ethanol administration for 2 h, with a 1-h pause between cycles, and we administrated a total of three cycles for her.

Anhydrous ethanol (total dose: 13.5 mL) was administered, and the sacral epidural catheter was removed 2 h after completing the ethanol infusion. No obvious changes were observed in neurological findings, such as bladder or rectum dysfunction after the three ethanol injections were completed.

NRS scores were assessed shortly after the ethanol block ended and again the following morning. The patient’s anal pain decreased to NRS 1 (at the end of the injection) and to 0 (the following morning). Her standing pain disappeared, her opioid dose was reduced, and overall pain management improved markedly.

She was discharged on the 5th day after the ethanol infusion and transitioned to home palliative care. Her pain remained controlled, and she passed away 41 days after discharge without further pain exacerbation.

## Discussion

Many patients with advanced cancer develop various symptoms that can limit the effectiveness of pain treatment and significantly impair quality of life [4]. A previous report indicated that pain treatment was inadequate for 14% of patients [4].

To date, there have been no reports of continuous sacral epidural ethanol injections specifically for anal pain resulting from intraperitoneal dissemination of malignant pheochromocytomas. Pheochromocytomas are rare catecholamine-secreting tumors with an incidence of approximately 2–8 per million adults, of which approximately 10% are malignant, and pain in these patients is typically caused by direct tumor invasion and multiple bone metastases [5].

Rectal cancer and peritoneal tumor dissemination often cause perineal and anal pain, which is highly resistant to opioids [2]. Opioids are generally effective for C-fiber-derived pain, but perineal and anal pain caused by contact with the tumor is thought to be primarily mediated by Aδ fibers [3, 6]. This type of pain often worsens during defecation or when sitting. Intrathecal phenol block is effective for pain management of these perineal pains [1, 2, 6]. However, its applicability is limited because it tends to cause vesicorectal and lower limb movement disorders, often requiring stoma extension and urinary catheterization [1, 5, 7].

A one-shot sacral epidural ethanol injection has been reported as a nerve-destructive method [8]. However, it has been reported that the pain relief effect of a single administration of ethanol is often inconsistent, with many patients requiring repeated blocks.

In contrast, the continuous sacral epidural ethanol block is a nerve destruction technique first reported by a Japanese anesthesiologist [3], blocking only the sensation at the S4 and S5 levels. This procedure can be performed safely by first administering a continuous dose of a local anesthetic [9].

In recent years, reports have highlighted the effectiveness of continuous sacral epidural blocks for managing anal pain resulting from rectal invasion by prostate cancer [9] and rectal tenesmus associated with cervical cancer [10].

Another advantage is that nerve destruction can be performed only in the S4 and S5 regions by administering a small amount of ethanol continuously through the epidural catheter. This method can provide pain relief without causing motor disorders of the lower limbs, bladder, or rectum.

Thus, sacral epidural continuous ethanol infusion therapy is considered appropriate for severe pain that is not relieved by the administration of analgesics such as opioids and NSAIDs [10].

The procedure for a continuous sacral epidural ethanol block is as follows [3]. After inserting the catheter into the sacral epidural space, continuous local anesthetic administration begins at 2 mL/h for approximately 12 h, reducing the infusion rate if urinary dysfunction or lower extremity sensorimotor nerve block occurs. If neurological findings such as dysuria or sensory loss in the lower extremities are observed, the continuous dosage is decreased by 0.5 mL/h. This sustained dose serves as a guideline for a safe, sustained dose of absolute ethanol.

Before continuous administration, the position of the catheter tip is confirmed by X-ray and contrast agent administration. After positioning is confirmed, 1.5 mL of absolute ethanol is slowly administered over approximately 5 min for nerve disruption, followed by a continuous ethanol infusion at the same rate as the initial anesthetic dose.

The administration was continued for 2 h, and the patient was paused for 1 h to check for changes in neurological findings. During the continuous administration of ethanol, the patient was allowed to assume a comfortable position on the bed. Similarly, during the 1-h break, there were no restrictions on the patient’s position, and they were encouraged to remain comfortable in bed.

However, given the risk of falling, it is advisable to limit patient ambulation, allowing movement only to a portable bedside toilet during the break. The above 3-h treatment was considered to be one course, which ends after 1–3 courses [3, 9, 10]. After the ethanol injection, the patient was allowed to walk if they did not feel uncomfortable or unsteady.

Although sacral epidural ethanol injection therapy carries a risk of vesicorectal disorders or lower extremity movement disorders, the incidence of these complications is generally considered to be low [3, 10]. This is because the optimal dosage can generally be estimated from a test block, and this dosage is believed to be unlikely to cause bladder or rectum disorders or neurological symptoms in the lower limbs. However, if symptoms such as bladder or rectum dysfunction or sensory loss in the lower limbs are observed, ethanol administration should be discontinued.

The advantage of this method is that it is a relatively simple procedure, as it can be performed at the bedside as well.

This method involves indwelling a catheter in the sacral epidural space and continuously administering a small amount of absolute ethanol to block sensation only in the perineal and anal areas and achieve pain relief. It is mainly suitable for patients who are able to excrete on their own because it can block the perception of S4 and S5 regions without affecting excretory or motor functions.

We report a case in which sacral epidural ethanol injection therapy was successful in managing anal pain that worsened while standing in a patient with multiple metastases from malignant pheochromocytoma. Given that sacral epidural ethanol injection carries a relatively low risk of complications, it is recommended for anal pain that is unresponsive to standard analgesic treatment.

## Data Availability

Not applicable.
